# Neurogenesis Drives Stimulus Decorrelation in a Model of the Olfactory Bulb

**DOI:** 10.1371/journal.pcbi.1002398

**Published:** 2012-03-15

**Authors:** Siu-Fai Chow, Stuart D. Wick, Hermann Riecke

**Affiliations:** 1 Engineering Sciences and Applied Mathematics, Northwestern University, Evanston, Illinois, United States of America; 2 Department of Physics, North Central College, Naperville, Illinois, United States of America; 3 Northwestern Institute on Complex Systems, Northwestern University, Evanston, Illinois, United States of America; École Normale Supérieure, College de France, CNRS, France

## Abstract

The reshaping and decorrelation of similar activity patterns by neuronal networks can enhance their discriminability, storage, and retrieval. How can such networks learn to decorrelate new complex patterns, as they arise in the olfactory system? Using a computational network model for the dominant neural populations of the olfactory bulb we show that fundamental aspects of the adult neurogenesis observed in the olfactory bulb – the persistent addition of new inhibitory granule cells to the network, their activity-dependent survival, and the reciprocal character of their synapses with the principal mitral cells – are sufficient to restructure the network and to alter its encoding of odor stimuli adaptively so as to reduce the correlations between the bulbar representations of similar stimuli. The decorrelation is quite robust with respect to various types of perturbations of the reciprocity. The model parsimoniously captures the experimentally observed role of neurogenesis in perceptual learning and the enhanced response of young granule cells to novel stimuli. Moreover, it makes specific predictions for the type of odor enrichment that should be effective in enhancing the ability of animals to discriminate similar odor mixtures.

## Author Summary

The olfactory bulb is one of only two brain regions in which new neurons are added persistently in substantial numbers even in adult animals. This leads to an ongoing turnover of interneurons, in particular of the inhibitory granule cells, which constitute the largest cell population of the olfactory bulb. The function of this adult neurogenesis in olfactory processing is only poorly understood. Experiments show that it contributes to perceptual learning. We present a basic computational model that is built on fundamental aspects of the granule cells and their connections with the excitatory mitral cells, which convey the olfactory information to higher brain areas. We show that neurogenesis can reshape the network connectivity in response to olfactory input so as to reduce the correlations between the bulbar representations of even highly similar stimuli. The neurogenetic adaptation of the stimulus representations provides a natural explanation of the perceptual learning and the different response of young and old granule cells to novel odors that have been observed in experiments. The model makes experimentally testable predictions for training protocols that enhance the discriminability of odor mixtures.

## Introduction

Contrast enhancement and decorrelation are common steps in information processing. They can reshape neuronal activity patterns so as to enhance down-stream processing like pattern discrimination, storage, and retrieval. The activity patterns can be complex and new patterns may become relevant due to changes in the environment or in the life circumstances of the animal. How can networks adapt to such demands, as they arise, for instance, in the olfactory system? What are neural substrates that would allow the necessary network restructuring?

In the olfactory system initial sensory processing is performed in the olfactory bulb. Its inputs consist of activation patterns of its 100–1,000 glomeruli, each of which can be considered as an individual input channel representing a specific olfactory receptive field. The bulbar network reshapes the patterns representing odor stimuli and typically reduces the correlation between output patterns representing similar odors as compared to the respective input patterns [1]–[3]. It does so despite the fact that even simple odors evoke complex activation patterns due to the fractured representation of the high-dimensional odor space on the two-dimensional glomerular surface [4]. Unlike spatial contrast enhancement in the retina [5], this decorrelation can therefore not arise from local lateral inhibition that is confined to neighboring glomeruli [3], [6]. What types of network connectivities can then underlie the enhancement of small, but significant differences in the representation of similar odors?

Previously, a number of different decorrelation mechanisms have been proposed, each of which exploiting a different aspect of the nonlinear dynamics of the bulbar network. The network connectivities were taken to be fixed, either without any lateral inhibition [4], with all-to-all inhibition [7], or with sparse random connections across large portions of the bulb [3]. These networks were shown to reduce quite effectively the correlation between the representations of moderately similar stimuli.

A different perspective is suggested by two distinctive features of the olfactory system: i) many odors do not have an intrinsic meaning to the animal and their significance is likely to be learned by experience [8]–[10]; ii) the bulbar network structure is not static but undergoes persistent turn-over due to neurogenesis and apoptosis even in adult animals [11], [12].

So far, the specific role of adult neurogenesis for olfactory processing is only poorly understood [13], [14]. It is known that environmental changes like sensory deprivation [15]–[18] and odor enrichment [19]–[21], associative learning [22]–[25], and life circumstances like mating [26] and pregnancy [27] affect anatomical and functional aspects of the olfactory bulb. Moreover, genetic [28], [29], pharmacological [30]–[32], and radiational manipulations [33], [34] have identified the significance of neurogenesis in these modifications.

Here we ask whether the neuronal turnover associated with adult neurogenesis can provide a neural substrate for the adaptation of the network to the decorrelation of different relevant stimuli that may be highly similar. Such a contribution of neurogenesis to pattern separation has been proposed for the olfactory bulb as well as the dentate gyrus [35]. We use a minimal computational network model of neurogenesis in the olfactory bulb that incorporates the persistent addition of new inhibitory interneurons (granule cells) into the olfactory bulb [36], their connection with the principal mitral cells via *reciprocal* synapses through which the mitral cells excite the granule cells and the granule cells inhibit the mitral cells [37], and the activity-dependent apoptosis of the granule cells [15], [32], [38]–[41]. Using stimulus ensembles based on glomerular excitation patterns observed in rat [42] we find that the networks learn to decorrelate even very similar stimuli. This results largely from the surviving granule cells detecting strongly co-active mitral cells and providing lateral inhibition between them. Our modeling gives a natural interpretation of recent experiments on the role of neurogenesis in the perceptual learning of a non-associative odor discrimination task [40] and the detection of novel odors [20]. Our computational model predicts that learning to decorrelate highly similar mixtures comprised of dissimilar components requires the exposure to a mixture of the components rather than the individual components themselves. This can be tested in behavioral experiments using suitable enrichment protocols [40], [43]–[45].

## Results

### Activity-Dependence of Survival Drives Decorrelation

**Figure 1:**
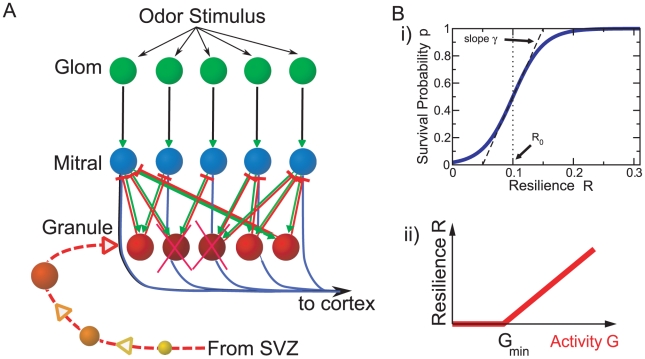
Main components of the model. **A**) Sketch of the recurrent bulbar network model with neurogenesis. Odor stimuli evoke glomerular activation patterns (green). The glomeruli drive mitral cells (blue), which relay the information to cortex. In addition, they excite granule cells (red), which through their reciprocal synapses provide self-inhibition and lateral inhibition to the mitral cells. New granule cells migrate persistently from the subventricular zone to the olfactory bulb and are incorporated into the network (yellow to orange). They are removed if their activity is too low (dark red). **B**) The survival probability of granule cells depends sigmoidally on their resilience (i), which is a threshold-linear function of their activity (ii), summed over the stimulus ensemble.

In our computational model we consider the recurrent network formed by principal mitral cells and inhibitory granule cells. We focus on the adaptive restructuring of the network connectivity in response to a stimulus ensemble and model the individual neurons in a minimal fashion using linear firing-rate dynamics (cf. *METHODS*, *Discrete Adaptive Network Model*). Focusing on the evolution of the network structure we ignore transients in the evolution of the neuronal activities and consider only their steady states in response to any given odor stimulus. The network is persistently rewired by adding in each time step of the network evolution randomly connected new granule cells and removing granule cells that are not sufficiently active during the steady state reached in response to odor stimulation (Fig. 1). Specifically, the survival probability of a granule cell depends in a sigmoidal fashion on its ‘resilience’ 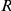, which we introduce as its thresholded activity summed over the stimulus ensemble.

**Figure 2:**
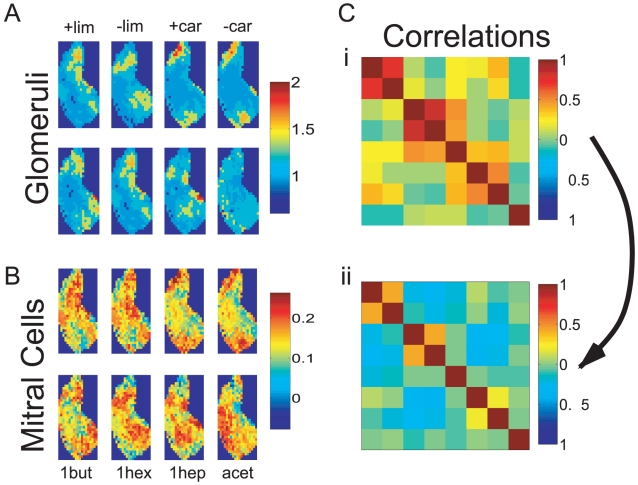
Decorrelation of natural stimuli. **A**) Glomerular activation patterns in rat for the odorants 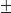-limonene, 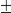-carvone, 1-butanol, 1-hexanol, 1-heptanol, and acetic acid [42]. **B**) Mitral cell activity patterns of a network trained on all eight stimuli. **C**) Correlation matrix of the input patterns (i) and of the mitral cell output patterns (ii). The stimuli are ordered as in A and B. Parameters: 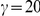, 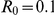, 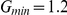, 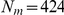, 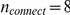, 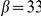, 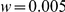, 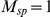.

In most of the computations we use input patterns that are based on a set of experimentally obtained glomerular activity patterns in rat [42] corresponding to the odorants 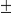-limonene, 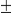-carvone, 1-butanol, 1-hexanol, 1-heptanol, and acetic acid (Fig. 2A). They drive 424 mitral cells, which in turn excite about 10,000 granule cells. Due to the reciprocal character of these synapses each granule cell provides self-inhibition to each of the eight mitral cells that drive it as well as lateral inhibition between them (Fig. 1A). All synaptic strengths are taken to be fixed. Unless noted otherwise, all excitatory and all inhibitory synapses have equal strengths, respectively.

The network that eventually emerges as a statistically steady state from the persistent rewiring substantially reshapes the representation of the stimuli (Fig. 2B). In particular, the mitral cell activation patterns, which represent the output of the olfactory bulb, differ from each other significantly more than the glomerular input patterns. To quantify this reduction in similarity we use the Pearson correlation 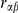 of the patterns associated with stimuli 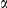 and 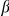 (cf. Eq.(10)), as has been done in previous, experimental studies [1]–[3]. Thus, the network achieves a substantial decorrelation of the stimulus representations (Fig. 2Ci,ii). This is the case for the highly similar 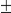-limonene- and 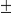-carvone-pairs as well as the less correlated, remaining stimuli of the odor ensemble. Moreover, through the enhanced inhibition of mitral cells that are strongly driven in this stimulus ensemble and the spontaneous activity of mitral cells that receive very little or no input [3], [46] the network reshapes the quite focal input patterns into output patterns in which the activity is more broadly distributed over the whole network (Fig. 2B). Such a reduction of the focality of the output patterns has been observed for mitral cell activity in zebrafish [3]. Particularly for stimuli that predominantly overlap in these focal areas such a reshaping of the pattern can reduce the correlation significantly.

**Figure 3:**
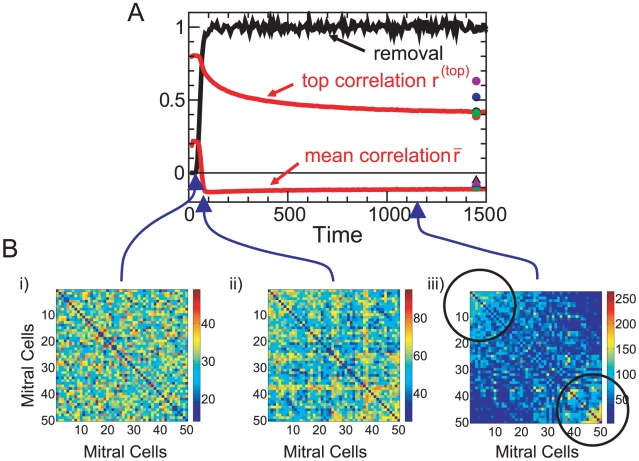
Decorrelation and connectivity. Evolution of the pattern correlation and rate of granule cell removal (scaled by their influx) (**A**), and the effective connectivity matrix 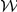 between pairs of mitral cells (cf. Eq.(7)) (**B**). Initially (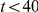) almost all granule cells survive, generating a random connectivity that does not decorrelate the stimuli (**Bi**). By 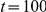 the selective removal of weakly active granule cells leads to a structured connectivity (**Bii**) that reduces the mean correlation 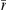. The highly similar stimuli 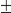-limonene and 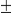-carvone are only decorrelated by strong inhibition between highly co-active mitral cells (marked by black circles), which emerges in the final steady state (**Biii**). Parameters for the simulation in **A** as in Fig. 2. The correlations have been averaged over 16 runs. The symbols at 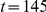
0 denote output correlations for different slopes of the survival curve, 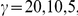
2.5,1 (cf. Fig. 1Bi). For visual clarity the connectivities are shown in **B** for a reduced network of 50 instead of 424 mitral cells (for parameters see Fig.S1B in Text S1). In the connectivity matrices the diagonal elements have been divided by 10.

Insight into the mechanisms underlying the decorrelation by the network is gained by following the evolution of the connectivity and the associated decorrelation performance as the network builds up from a network without any granule cells (Fig. 3). The early stages of this evolution are not meant to mimic the peri-natal development of the bulb, which is controlled by mechanisms other than those included in this model. To visualize the network connectivities the stimuli are down-sampled to 50 channels (cf. *METHODS*, *Natural Stimuli*) and the two-dimensional activation patterns are re-arranged into one-dimensional vectors in which the mitral cells that are strongly activated during the 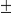-limonene presentation are located at the beginning of the vector and those that dominate during the 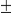-carvone presentation at the end. Because the overlap between the activation patterns of these two pairs of enantiomers is small there are only few mitral cells that receive significant input for both types of stimuli. They end up towards the middle of the activity vector. For visual clarity the diagonal elements of the connectivity matrices are divided by 10.

During the initial phase 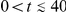 the granule cell population is small and provides only little inhibition to the mitral cells. Their activities and with them the activities of the granule cells are therefore high and none of the granule cells are removed (Fig. 3A). Since the granule cells establish random connections with the mitral cells the resulting effective connectivity between the mitral cells is essentially random (Fig. 3Bi) and the activity patterns are only reduced in amplitude without any qualitative changes; the correlations remain high. As the mitral cell activities decrease, some granule cells fall in their activity and resilience below the soft survival threshold 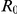 (cf. Fig. 1Bi) and their survival probability drops drastically (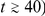. This apoptosis is selective, resulting in a structured connectivity, in which more highly active mitral cells receive stronger inhibition (Fig. 3Bii), and a reduction of the mean pattern correlation. The correlation between the highly similar stimuli is, however, still high. In the third phase of the network evolution the size of the granule cell population remains constant, but the connectivity evolves slowly towards establishing strong effective mutual inhibition between mitral cells that are highly co-active during 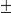-limonene or 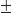-carvone presentations (marked by circles in Fig. 3Biii). In parallel, the correlation 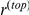 of these highly similar enantiomers is strongly reduced.

**Figure 4:**
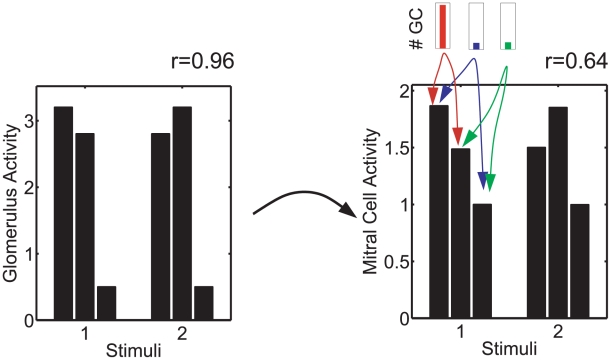
Decorrelation by inhibition of strongly co-active mitral cells. Since mitral cells 1 and 2 are strongly driven in both stimuli the population of granule cells (GC) connected to these mitral cells (red) is much larger than the other two populations (blue, green). The associated inhibition strongly suppresses the activities of mitral cells 1 and 2, but not of mitral cell 3, which reduces the correlation of the patterns from 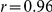 to 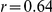. The mitral cells have a spontaneous activity 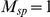.

The effectiveness of the inhibition of highly co-active mitral cells in decorrelating activity patterns is illustrated using a very simple example with stimuli exciting only three mitral cells (the relevant two stimuli are shown in Fig. 4). Like the highly similar olfactory stimuli in Fig. 2, these stimuli overlap in strongly co-active glomeruli. This allows the population of granule cells that connect to the mitral cells driven by these glomeruli to be much larger than the other two populations. The reciprocity of the synapses implies that these mitral cells receive substantially stronger inhibition than the other mitral cell. The resulting reduction in amplitude reduces also the correlation between the two mitral cell activity patterns. In Fig. 3Biii the corresponding enhanced connectivity between mitral cells that are highly co-active during 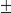-limonene (or 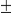-carvone) stimulation is marked by black circles.

### Threshold Promotes Lateral Inhibition Based on Co-Activity

What determines the performance of the networks arising from the persistent turn-over? The granule-cell survival is controlled by two thresholds: i) for each stimulus for which the granule cell activity surpasses the resilience threshold 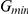 its resilience 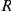 increases (cf. Eq.(8)) and ii) the resilience accumulated across all stimuli of the ensemble has to be above the soft survival threshold 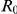 in order for the granule cell to have a significant survival probability (cf. Eq.(9)). The survival threshold 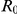 controls in particular the total number of granule cells and with it the overall level of inhibition. In general, the overall correlation of the outputs decreases with increasing inhibition (data not shown) at the expense of the output amplitudes. In our comparisons we adjust therefore 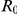 to keep the mean output amplitudes fixed.

**Figure 5:**
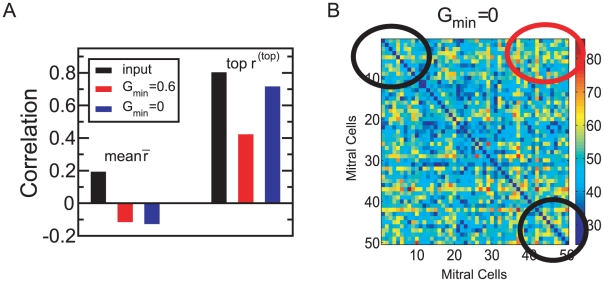
Resilience threshold 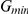 reduces interference and enhances decorrelation of highly correlated stimuli. **A**) For 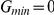 the networks achieve the same level of overall decorrelation as networks with suitable 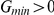, but they decorrelate the representations of highly similar stimuli very poorly. Parameters: 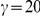, 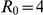, 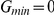, 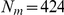, 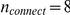, 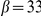, 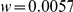, 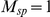. For 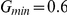 parameters as in Fig. 2. **B**) Effective connectivity matrix 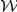 for 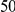 mitral cells with diagonal elements divided by 10. For 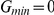 the interfering connections between mitral cells that are active for 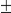-limonene *or* for 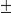-carvone (red ellipse) are as strong as those between co-active cells (black ellipses); cf. panel bottom right on Fig. 3. Parameters: 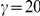, 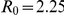, 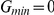, 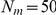, 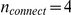, 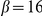, 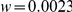, 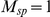.

A more subtle and interesting role is played by the resilience threshold 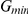. For 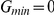 the network achieves an overall decorrelation that is quite comparable to that of the network of Fig. 2 with 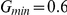; the highly similar stimuli 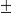-limonene and 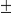-carvone, however, are only very poorly decorrelated (Fig. 5A). The origin of this poor performance is apparent in the effective connectivity obtained with 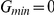 (Fig. 5B). A comparison with the connectivity arising for 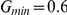 (Fig. 3) reveals that the connections among the mitral cells that are co-active in response to 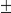-limonene (or 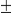-carvone) stimulation (black circles) are not stronger than among mitral cells that are not co-active (red circle). As had been observed in Fig. 3, it is the connections among co-active mitral cells, however, that are essential for decorrelating these stimulus representations.

**Figure 6:**
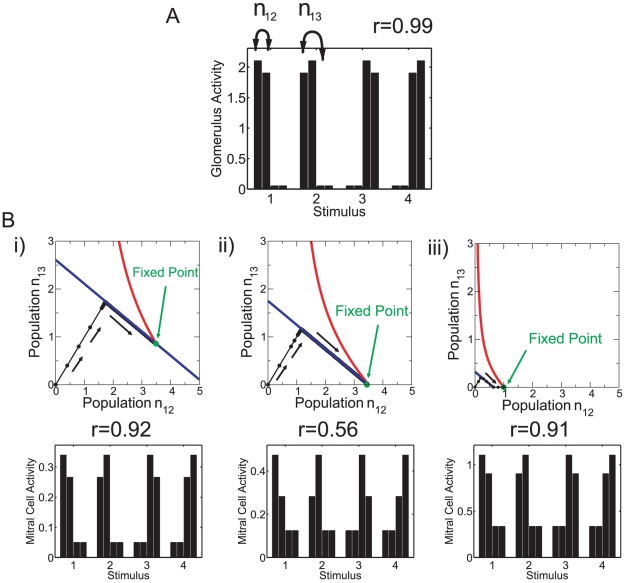
Interference and optimal resilience threshold 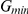. **A**) Two pairs of symmetrically related stimuli comprised of four glomeruli each (cf. eqs.(18,19). The granule cells are described by the populations 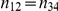 and 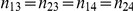. Stimulus pairs 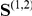 and 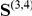 are highly correlated (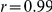). **B**) Top panels: Phase plane with nullclines 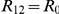 (red) and 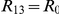 (blue) and the trajectory 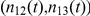 (black symbols) starting from 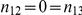 and ending up on the fixed point. The network evolution is indicated by black arrows. Bottom panels: mitral cell activity patterns. i) 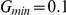. Interference (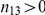) strongly suppresses the weakly driven mitral cells. High correlation (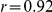). ii) 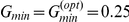. No interference (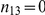), but strong inhibition among the highly co-active mitral cells through large population 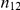. Low correlation (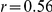). iii) 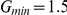. The inhibition of co-active mitral cells is weak. High correlation (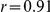). Other parameters: 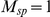, 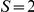, 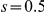, 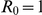, 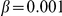, 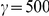.

How does the threshold 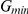 provide a co-activity detector? Why do the connections among mitral cells that are not co-active interfere with the decorrelation? The function of the threshold can be illustrated with a minimal set of two pairs of strongly correlated stimuli 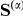 activating four glomeruli, 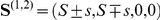 and 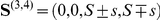 with 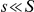 (Fig. 6A). Stimuli 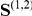 and 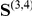 may be viewed as caricatures of the limonene and carvone enantiomers, respectively. The granule cells in population 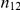 inhibit the mitral cells that are co-active in stimuli 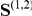 (cf. Fig. 6A) and are therefore needed for decorrelation. The granule cells in population 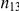, however, are connected to mitral cells that are not co-active in any of the stimuli; they may interfere with the performance of the network. The resilience 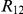 of the granule cells in population 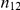 is comprised of two large contributions due to the strong inputs in stimuli 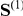 and 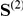 and two small contributions from stimuli 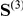 and 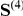, while the resilience 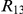 of the cells in population 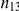 is determined by 4 intermediate contributions. In our model (3,4) for the neuronal dynamics the granule cell activities are linear in the mitral cell activities. For 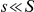 the activity 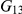 of the interfering population 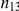 is almost the same for all four stimuli and is close to the average of the activity 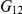 across the four stimuli. As a result, the rectifier, which makes the resilience function (8) concave, renders the granule cells that establish interfering connections less resilient than the granule cells connecting co-active mitral cells, 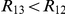. This suppresses the interfering population 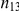 relative to 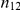, as is apparent in a comparison of Fig. 3Biii and Fig. 5B.

Within the framework of the population formulation eqs.(15,16,17) the simplicity of the minimal stimulus set of Fig. 6A allows a detailed analysis of the role of the threshold in the balance between the suppression of interfering connections and a reduction of the beneficial inhibition of co-active mitral cells. Due to the symmetry of the stimulus ensemble only two granule-cell populations have to be analyzed, 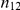 and 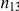. Their dynamics can be understood using a phase-plane analysis. For steep survival curves 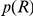 the nullclines of 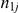, which are defined by 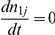, are very well approximated by 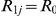 (cf. Fig. 1Bi). Starting from 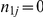, both population sizes increase linearly in time until they reach one of the two nullclines. Then the system follows slowly that nullcline until a fixed point is reached. This can be the intersection of the two nullclines (Fig. 6Bi). In addition, since 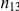 cannot become negative, an intersection of the nullcline 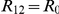 with the axis 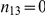 also represents a fixed point if at that point 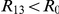 (Fig. 6Biii) and similarly with the roles of 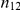 and 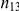 interchanged.

A straightforward expansion shows that for highly similar stimuli, 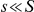, the correlation between the two output patterns 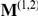 is given by
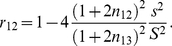
(1)Thus, as expected, the correlation decreases with increasing reciprocal inhibition 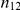 of co-active mitral cells and increases with increasing strength of the interfering connections 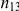. As discussed above, the relation between these two populations can be controlled using the threshold 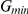. For fixed resilience threshold 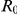 the correlation is minimized for (cf. Eqs.(23,22))
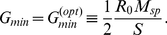
(2)This is the smallest value of 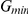 for which the interfering connections vanish, 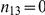. Thus, it maximizes the inhibition between co-active mitral cells without inducing interference. This leads to optimal decorrelation, as is also apparent in the output activity patterns in the bottom panels of Fig. 6B.

Thus, the threshold 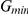 in the resilience suppresses interfering connections between mitral cells that are not co-active and promotes a connectivity that is based on co-activity. To provide a context of the performance of this co-activity based connectivity we compare the decorrelation achieved by the resulting networks with that obtained by a number of other types of adaptive networks. In some of them the inhibition is also based on co-activity, in others on distance, correlation, or covariance (see Text S1 with Figs.S1,S2,S3 therein). We find that the networks whose adaptation mechanism is based on some form of co-activity of mitral cells or glomeruli are able to decorrelate representations of highly similar stimuli and achieve a reduction of the overall correlations without and with significant spontaneous mitral cell activity. Among these networks are networks motivated by an earlier model for neurogenesis [47] as well as networks that aim to orthogonalize the stimulus representations by orthogonalizing (and normalizing) the activity vectors of pairs of mitral cells [48]. Alternatively, the connectivities can also be based on the correlations or covariances of the inputs. For instance, a correlation-based connectivity was found to capture the outputs of the bee antennal lobe, which is the insect homolog of the olfactory bulb, better than random or local connectivities [49]. We find that correlation- and covariance-based recurrent networks do not decorrelate stimulus representations very well. In various situations they even tend to increase rather than decrease the correlations. This reflects, in part, the fact that they are not sensitive to the spontaneous activity of the mitral cells.

### Imperfect Reciprocity of Synapses Is Sufficient

Anatomically, the dendrodendritic synapses between mitral cells and granule cells are found to be predominantly reciprocal, i.e. each granule cell has inhibitory connections only to those mitral cells from which it receives excitatory connections [37]. In combination with the threshold 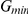 this establishes effectively inhibitory lateral connections selectively between highly co-active mitral cells and allows the networks to decorrelate their highly correlated inputs.

As implemented in our model so far, the reciprocal synapses not only provide an anatomical connection between co-active mitral cells but due to the homogeneity of the inhibitory synaptic weights they also induce a symmetric connectivity matrix and the amount of self-inhibition that a given mitral cell experiences is directly related to the amount of lateral inhibition it provides to other mitral cells. What roles do these different aspects play in the decorrelation?

**Figure 7:**
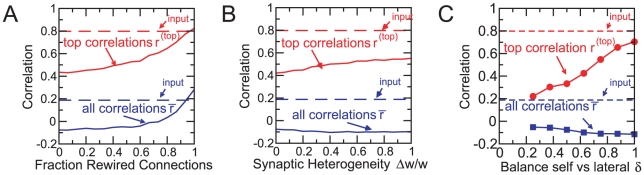
Effective decorrelation does not require complete reciprocity of the synapses. **A**) A fraction of the inhibitory connections are rewired to a randomly chosen mitral cell. Dashed lines denote input correlations. **B**) The inhibitory synaptic strengths are picked with equal probability from the two values 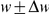. **C**) Reducing self-inhibition in favor of lateral inhibition, 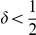, enhances the decorrelation. Parameters: 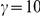, 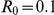, 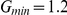, 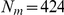, 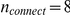, 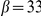, 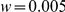, 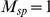.

To test the importance of the correct anatomical connections we redirect a fraction of the inhibitory connections of each granule cell to randomly chosen mitral cells instead of the mitral cells that drive that granule cell. As expected, as the fraction of such non-reciprocal synapses increases the correlations increase as well. Without any reciprocal synapses the network does not decorrelate the stimuli at all (Fig. 7). The network performance is, however, quite robust: the overall decorrelation deteriorates noticeably only when more than 50% of the connections have been rewired. The highly correlated stimuli are, however, more sensitive to the rewiring with 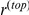 increasing from 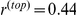 to 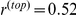 when 50% of the connections are rewired, while 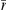 changes only from 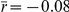 to 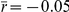.

The granule cells deliver their inhibitory inputs onto the secondary dendrites of the mitral cells at highly variable distances from the mitral cell somata. Their effect on the mitral cell firing will therefore vary over quite some range; in fact, some synaptic contacts will be too far away from the mitral cell soma to have any noticeably effect on that mitral cell's firing. To assess the impact of such heterogeneities we modify the inhibitory synaptic weights, which so far had the same value 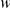 for all synapses, by picking them with equal probability from the two values 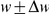. This breaks the symmetry of the inhibition and for 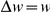 half of the inhibitory connections are completely ineffective. The overall decorrelation is, however, not affected by this heterogeneity and even the decorrelation of the highly similar stimuli deteriorates only slightly over the whole possible range 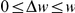 (Fig. 7B). Essentially the same result is obtained if the synaptic strengths are distributed uniformly in the interval 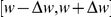. While for very large granule cell populations the heterogeneities of different granule cells are expected to average out each other, for the parameters used in our study the effective connectivity matrix is still noticeably asymmetric: its anti-symmetric component amounts to about 20% of the symmetric one.

Through the reciprocal character of the dendrodendritic synapse a granule cell mediates lateral inhibition between the mitral cells that drive it as well as self-inhibition of each of them. Due to the complex dendritic dynamics of granule cells [50], [51] these two types of inhibition can be of different strength. In fact, recent observations suggest that self-inhibition is significantly weaker than lateral inhibition [52]. While our minimal model does not capture any explicit dendritic processing, the strength of self-inhibition and lateral inhibition that a mitral cell receives is given by the diagonal and off-diagonal coefficients of the effective connectivity matrix 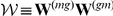, respectively. We can therefore change the balance between self-inhibition and lateral inhibition phenomenologically by rescaling the diagonal terms, 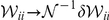 with 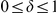, at the expense of the off-diagonal terms, 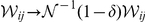 for 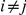, while keeping the row-sum of the matrix fixed through the normalizing factor 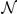. Reducing self-inhibition in this fashion (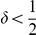) enhances the decorrelation of the representations of the natural stimuli significantly (Fig. 7C), because it further enhances the competition between dominant, co-active mitral cells. Conversely, increasing the self-inhibition (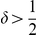) reduces the competition. In the complete absence of lateral inhibition (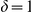) granule cells are effectively coupled only to a single mitral cell. This provides still good overall decorrelation, but the representations of the highly similar odors are only poorly decorrelated. Thus, the experimentally observed reduction of self-inhibition may contribute to an improved decorrelation performance of the bulbar network.

These comparisons show that for effective decorrelation the most important aspect of the reciprocity of the dendrodendritic synapse is that it provides mutual anatomical connections between the relevant mitral cells, i.e. between those that are co-active for some stimuli. The effective synaptic strengths can be quite heterogeneous without compromising the performance of the network. In fact, reduced self-inhibition can enhance the decorrelation substantially.

### Young Granule Cells Respond to Novel Odors

One possible role of neurogenesis is to provide a persistent supply of new neurons, which may play a different role than old, mature neurons. An aspect of this type has been identified in experiments focusing on the responsiveness of young and old adult-born granule cells [20], [53]. In the experiments, adult-born precursor cells, which develop into granule cells, were marked in the subventricular zone. After they have migrated to the olfactory bulb and have integrated into the bulbar network their response to odor stimulation was measured using the expression levels of various immediate early genes. It was found that the fraction of adult-born granule cells that respond to novel odors is significantly higher shortly after their arrival in the olfactory bulb than a few weeks later. It has been argued therefore that one important function of the young granule cells may be to serve as novelty detectors [20].

**Figure 8:**
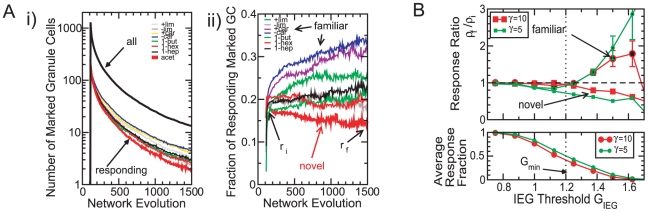
Young granule cells show enhanced response to novel odors. **A**) **i**) Granule cells are marked at 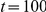. The total number of marked cells (black thick line) and the number of marked cells responding to one of the eight stimuli decreases with time. The stimulus ensemble consists of 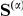, 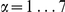. Stimulus 8 (acetic acid) is novel (cf. Fig. 2). **ii**) The fraction 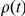 of marked granule cells that respond to the novel stimulus decreases with time. For the familiar stimuli it mostly increases. Parameters: 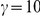, 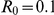, 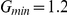, 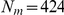, 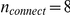, 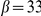, 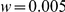, 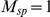, 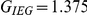. **B**) The IEG-activation threshold 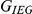 has to be close to the resilience threshold 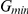. Bottom panel: for 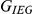 well above 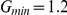 (dotted line) very few marked cells reach an activity above 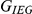 and are considered as responding to the stimuli. Top panel: For 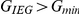 the response fraction 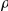 decreases with time for the novel stimulus (ratio of response fractions 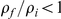, cf. panel Aii), while it tends to increase for the familiar stimuli (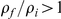, error bars denote standard deviation across the stimuli 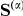, 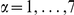). Parameters as in **A** except for the steepness 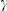 of the survival curve (cf. Fig. 1Bi), 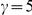 (green, small symbols), 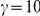 (red, large symbols). The results represent an average across 32 runs.

In our computational model a differential response of young and older adult-born granule cells to novel odors arises quite naturally. After establishing a network by exposing the system to the stimulus ensemble 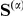, 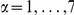, we mark granule cells as they are integrated into the network and measure their response to various stimuli as a function of their age. Assuming that the granule cell activity has to surpass a minimal value to activate the expression of the immediate early genes, we consider granule cells as responding if they reach an activity above a threshold 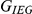. As the network evolves the less active granule cells die and are removed from the network (Fig. 8Ai). As in the experiments, we find that the fraction of young adult-born granule cells that respond to a novel stimulus, i.e. a stimulus that is quite different from the stimuli in the background ensemble, decreases as the granule cells become older (Fig. 8Aii). This decrease results from the reduced survival probability of these cells, which is due to the weak drive they receive by the stimuli in the stimulus ensemble that determines granule-cell survival. In contrast, the fraction of granule cells that respond to a familiar stimulus, i.e. a stimulus in the background ensemble, decays very little or even increases over the same time frame, reflecting their higher survival rate.

For what range of the threshold 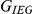 does our model yield results that agree qualitatively with the experiments in [20]? When the threshold 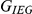 is increased beyond the resilience threshold 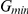 ever fewer marked granule cells respond and the fraction of marked granule cells that respond to the stimuli - averaged over all stimuli - drops from 1 to 0 (Fig. 8B bottom panel). Thus, the experimentally obtained response fractions of 10–20% [20] set an upper limit for 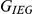 relative to 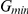. At the same time, decreasing 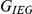 reduces the difference between the temporal evolution of the response to novel and to familiar stimuli. We characterize the evolution by the ratio 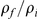 between the fraction 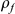 of granule cells responding to the stimulus at the final time of the simulation and the fraction 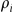 immediately after the end of the marking period. On average this ratio increases with increasing 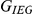 for the familiar stimuli, but it decreases for the novel stimulus (Fig. 8B top panel). For the response to novel odors to differ significantly from that to familiar odors 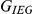 cannot be much smaller than 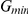. It is worth noting that varying the steepness 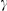 of the survival curve does not affect the decorrelation of the odor stimuli substantially (symbols at 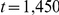 in the top panel of Fig. 3), but the difference in the response to novel compared to familiar odors is significant only if the survival curve is not too steep (Fig. 8B top panel).

Thus, the activity-dependent survival of the granule cells combined with their random connections to the mitral cells is sufficient to capture the experimentally observed enhanced response of young adult-born cells to novel stimuli if the threshold 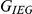 for the activation of the immediate early genes is close to the resilience threshold 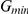, which is an essential determinant of the survival of the granule cells.

### Neurogenesis Contributes to Perceptual Learning

In a wide range of experiments possible connections between adult neurogenesis and animal performance have been investigated employing various tests of odor detection, odor discrimination, short-term and long-term memory, and fear conditioning [19], [29]–[34], [40], [43]–[45]. No simple picture regarding the role of neurogenesis in odor discrimination and odor memory has, however, emerged so far. This may in part be due to the fact that higher brain areas are likely involved in many of the behavioral tasks; they may well compensate for some changes occurring in the olfactory bulb and therefore possibly mask certain effects of the neurogenesis.

A behavioral task that may reflect bulbar odor representations relatively directly is the spontaneous, non-associative odor discrimination based on habituation, which has been shown to result predominantly from bulbar processes [54]–[56]. These experiments exploit the decreasing interest an animal typically displays to repetitions of the same stimulus: the animal's response to a second stimulus after if has habituated to a first stimulus is a measure of the degree to which the animal discriminates the two stimuli [55]. Exposing animals to extended periods during which their environment is enriched with additional odors enhances their spontaneous odor discrimination [40], [43]–[45]. This is indicative of perceptual learning. The dominance of bulbar processing in this task [54]–[56] suggests that the enrichment induces changes in the bulbar odor representations [56]. Since the enhancement is significantly suppressed if neurogenesis is halted pharmacologically [40], it is likely that the changes in the odor representations reflect a restructuring of the bulbar network. Importantly, for the enrichment to improve the performance the odors employed have to be related to the odors that are to be discriminated [43].

**Figure 9:**
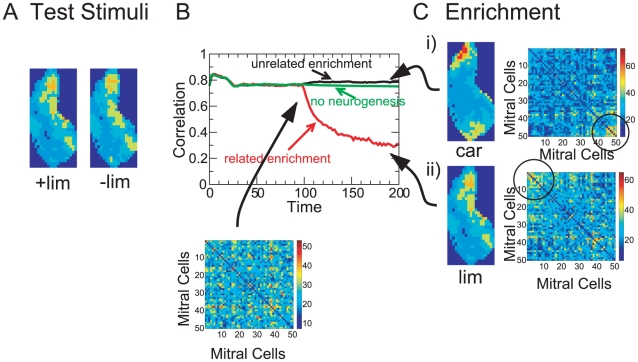
Perceptual learning. Correlation (**B**) of the test stimuli 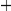-limonene and 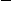-limonene (shown in **A**) as a function of time. Enrichment, beginning at 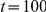, changes the connectivity. Enrichment with the related odors 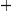-limonene and 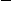-limonene (**Cii**, only 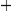-limonene is shown) strongly reduces the correlation, whereas enrichment with the unrelated odors 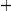-carvone and 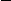-carvone (**Ci**, only 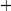-carvone shown) does not. Enrichment with a related odor but without neurogenesis does not enhance the decorrelation. Parameters: 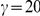, 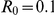, 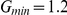, 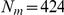, 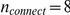, 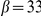, 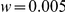, 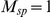. Background stimuli: 1-butanol, 1-hexanol, 1-heptanol, and acetic acid.

The perceptual learning observed in the experiments is captured in our minimal computational model. We use an ensemble of background stimuli, which establishes a default network connectivity, and test the performance of the network with two test stimuli (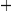-limonene and 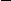-limonene). They are not included in the stimulus ensemble that drives the network evolution. For the default network the correlation between the representations of the test stimuli is high, consistent with the fact that naive animals do not discriminate these odors spontaneously. Then the stimulus ensemble is enriched with additional odors (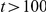). As the network adapts and evolves to a new steady state characterized by different effective connectivity matrices (Fig. 9C, right panels), the correlation between the two test stimuli evolves as well. If the odors used for the enrichment have sufficient overlap with the test odors the correlation between the test odors decreases substantially (red line in Fig. 9B). However, if the enrichment odors are only weakly related to the test odors the correlation of the test odors does not decrease (black line in Fig. 9B). In fact, in some cases the correlation between the test odors can even increase. As expected, if the influx of new granule cells is stopped with the onset of the enrichment the odor representations and their correlations are unaffected by the enrichment, even if the enrichment odor is close to the test odor (green line).

### Effective Enrichment: Overall Overlap Is Not Sufficient

In experiments, odor enrichment enhances the ability of the animals to discriminate similar odors only if there is sufficient overlap between the activation patterns of the stimuli used in the enrichment and those of the stimuli to be discriminated [43]. Our network model allows more specific predictions for the type of enrichment protocols that should be effective in enhancing the ability of the animals to discriminate a given set of test odors.

**Figure 10:**
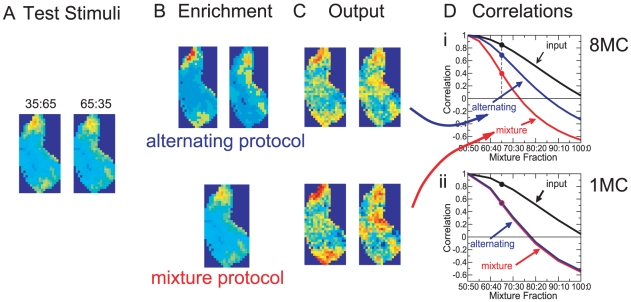
Effect of enrichment protocol on the decorrelation of similar mixtures. **A**) Sample test stimuli: mixtures of 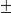
–limonene and 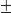
–carvone with mixture fractions 35∶65 and 65∶35. **B**) Enrichment stimuli. i) 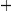-limonene, 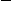-limonene, 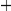-carvone, and 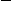-carvone alternating (only 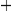-carvone and 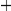-limonene shown), ii) 50∶50 mixture of 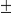-limonene and 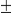-carvone. **C**) Output patterns for the test stimuli shown in A. Parameters as in Di. **D**) Correlations of mitral cell activities for the test stimuli as a function of the mixture fraction. i) Eight connections per granule cell. The mixture protocol achieves substantially better decorrelation than the alternating protocol. Parameters: 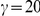, 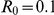, 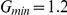, 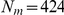, 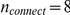, 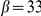, 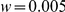, 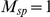. ii) One connection per granule cell. No significant difference between the protocols. Parameters: 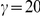, 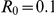, 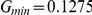, 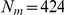, 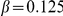, 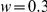, 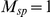.

We consider the decorrelation of very similar mixtures comprised of dissimilar components. Specifically, we use as components limonene (50% 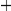
–limonene and 50% 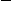
–limonene) and carvone (also both enantiomers in equal proportions), whose activation patterns have very little overlap (Fig. 2A). We employ two different enrichment protocols. In the first one pure limonene and pure carvone are added to a background of alcohols and acetic acid in an alternating fashion (Fig. 10B, top panel). Experimentally, this would correspond to presenting limonene and carvone separately at different times. In the second protocol an equal mixture of limonene and carvone is added to the background ensemble (Fig. 10B, bottom panel). In both protocols the activity-dependent removal of interneurons occurs only after the complete set of background and enrichment stimuli has been presented. To implement the mixtures in the model we assume that the glomerular activation patterns for mixtures are approximated sufficiently well by a linear combination of the patterns for the individual components.

While using the pure components in the enrichment decreases the correlation between the representations of the mixtures at all mixture ratios (‘alternating’ in Fig. 10Di) it does so substantially less than the network enriched with the 50∶50 mixture (‘mixture’ in Fig. 10Di). The stronger decorrelation obtained with the mixture protocol compared to the alternating protocol can also be recognized directly in the output patterns (Fig. 10C, bottom vs. top panel). This substantial difference arises because the decorrelation of the mixtures is strongly enhanced by mutual inhibition between mitral cells that are driven by limonene as well as carvone. This inhibition is provided by ‘mixed’ granule cells. By contrast, ‘pure’ granule cells are connected to mitral cells that are activated (almost) exclusively by limonene *or* carvone. As discussed in Sec. *Threshold Promotes Lateral Inhibition Based on Co-Activity*, in the context of interference, in the alternating protocol the mixed granule cells have a lower survival probability than the pure granule cells. In the mixture protocol, however, both types of granule cells have very similar survival probabilities. To assess the inhibition provided by these populations we consider the sum of the synaptic weights in the four quadrants of the effective connectivity matrix 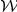 (cf. Fig. 3Biii). We find that the inhibition provided by the mixed granule cells in the mixture protocol is stronger than in the alternating protocol (see (27)). Insight into what controls these populations can be gained by considering again the simple caricature of Fig. 6A. Within that framework the mixture protocol can be viewed as a stimulus set in which all four glomeruli receive essentially equal input. Both types of granule cells have then equal survival rates. Within that model it is easily seen that the size of their populations falls between that of the mixed granule cells and the pure granule cells in the alternating protocol because the total resilience of the mixed granule cells has to be the same in both protocols (see Sec. *METHODS*, *Alternating vs Mixture Protocol*). Thus, compared to the alternating protocol the mixture procotol enhances the relevant inhibition and improves the decorrelation of the limonene-carvone mixtures.

If neurogenesis were to affect only interneurons that provide non-topographic inhibition and no lateral inhibition [4] both enrichment protocols would be expected to lead to the same level of decorrelation. Specifically, if in the model each granule cell makes only connections with a single mitral cell the alternating protocol leads to the same decorrelation of the limonene-carvone mixtures as the mixture protocol (Fig. 10Dii). Comparing the influence of the two enrichment protocols on the animals' ability to discriminate such mixtures may therefore give insight into the type of neurogenesis-dependent connectivity that dominates the decorrelation mechanism.

Thus, even though in both protocols the enrichment odors - taken together - have the same overlap with the test odors the model predicts that enrichment with the mixture protocol achieves substantially better decorrelation of the test stimuli than the alternating protocol.

## Discussion

To investigate the functional implications of the experimentally observed persistent turnover of inhibitory interneurons on sensory processing by the olfactory bulb we have used a minimal computational network model. The experimental observations forming the basis of our model are the reciprocity of the synapses between the interneurons (granule cells) and the principal neurons (mitral cells) [37] and the activity-dependent survival of adult-born granule cells [41]. In the model we have focused on the input from the mitral cells via the dendro-dendritic synapses as the dominant input controlling the activity and survival of the granule cells. Assuming in addition that the new cells connect to an essentially random set of mitral cells allows the model to capture parsimoniously various experimental observations and to make specific predictions.

### Novelty Detection

It has been observed that young granule cells are more likely than mature ones to respond to odors that are novel for the animal [20], [53]. This has been interpreted as a mechanism for novelty detection. Our model captures the enhanced response of young cells in a natural way. Since granule cells that respond to novel odors but not to the odors in the ongoing environment receive only little ongoing input, they do not survive for a long time and the fraction of granule cells responding to the novel odor decreases with their age. Thus, the observation of an enhanced response of young granule cells to novel odors suggests that new granule cells do not have a strong bias towards connecting to highly active mitral cells but connect also to mitral cells that have only been weakly active in the past. Such a strategy enables the network to learn to process novel odors.

Experimentally, the response of the granule cells was measured in terms of the expression of various immediate early genes (c-fos, c-jun, EGR-1/zif-268). The fraction of granule cells responding to the novel odors was found to be 10–25% for young cells and lower for older cells [20], [53]. Such an intermediate response fraction is obtained in our model if the threshold for the expression of the immediate early genes is close to that for the survival of the granule cells. This is suggestive of a common step in the pathways controlling IEG-expression and cell survival.

### Threshold Enhances Inhibition between Co-active Mitral Cells and Reduces Interference

The decorrelation of highly similar stimuli like the two pairs of enantiomers used in our computation hinges upon the presence of an activity threshold that the granule cells have to surpass to increase their survival probability. It enhances the connections between mitral cells that are highly active simultaneously and suppresses those between mitral cells that are strongly active albeit only in response to different stimuli.

Biophysically, a threshold for the survival of the granule cells may arise from the need to drive L-type Ca channels, which activate the MAPK pathway that leads to the stimulation of genes that are essential for neuronal survival [57], [58].

With the strengthening of inhibition between co-active mitral cells the mechanism underlying the adaptation in our model is somewhat related to that underlying other adaptive networks that have been studied previously. In an early neurogenesis model for the olfactory bulb the evolution of the effective pairwise inhibition between mitral cells was based directly on the scalar product of the mitral cell activities [47]. Adaptive networks that aim to orthogonalize the stimulus representations can do so via a connectivity that is based on the pairwise scalar products of input activities [48]. A somewhat different adaptive connectivity has been suggested in a modeling study of the bee antennal lobe. There it was found that a connectivity in which the inhibition is proportional to the correlations between the glomerular activities was able to match the observed output patterns better than random or local center-surround connectivities [49]. We have compared a few types of networks that exploit different adaptation algorithms and find that connectivities that are based on the co-activity of mitral cells or glomeruli achieve significantly better decorrelation than networks based on the correlations or covariances of the inputs. A particular problem of the latter algorithms is that they are not sensitive to mean activities of the cells and do not take the spontaneous activity of the mitral cells adequately into account.

### Reciprocity of Connections

An anatomically characteristic feature of the olfactory bulb is the reciprocal nature of the dendrodendritic synapses between mitral and granule cells. The purpose of this reciprocity is not well understood. Our computational modeling shows that it can play an essential role in exploiting the activity-dependent survival of the granule cells to establish a connectivity whose lateral inhibition reflects the co-activity of the mitral cells. This provides a mechanism for the network to learn to decorrelate even highly similar stimuli.

Biologically, the reciprocity may be imperfect in a number of ways. In principle, an inhibitory synapse could connect the granule cell to a mitral cell that is not the origin of the associated excitatory synapse. Modeling such a situation by a random rewiring of a fraction of inhibitory connections we find that the network performance is reasonably robust to such perturbations. However, when more than 50% of the synapses are rewired the performance deteriorates significantly and without any reciprocity the stimulus representations are not decorrelated at all.

A second type of imperfection of the reciprocity is likely to arise if the dendrodendritic synapse is located far from the soma of the mitral cell. In such a case the inhibition exerted by the granule cell may not have much effect on the mitral cell firing, although the granule cell is driven strongly by that mitral cell. This asymmetry can arise because excitation is driven by action potentials, which can travel long distances along the dendrite, whereas the shunting provided by the inhibition is confined to a distance comparable to the electrotonic length of the dendrite [59]. Thus, the effective inhibitory strength may vary substantially between synapses depending on their location relative to the soma. Mimicking such a heterogeneity by random variations in the synaptic strength we find that the network performance is only moderately affected by such effects. Since mitral cells are connected to many granule cells the heterogeneity of the combined synaptic strengths is likely to be reduced compared to the heterogeneities within individual granule cells. Such an averaging may be reduced if correlations between the strengths of different synapses, which may arise due to correlations in the physical distances between the cells, should be significant.

The reciprocity may also be perturbed because the strength of the self-inhibition that a mitral cell experiences on account of a given granule cell may differ from that of the lateral inhibition that said granule cell provides to other mitral cells. In fact, recent experiments suggest that self-inhibition is significantly weaker than lateral inhibition [52]. One cause for this difference may be the complex physiology of granule cells, which includes local dendritic calcium signaling, dendritic calcium spikes, and action potentials driven by sodium conductances [51]. Our minimal single-compartment model for the granule cell does not allow to capture these rich dynamics. However, on a phenomenological level the balance between self-inhibition and lateral inhibition can be modified by rescaling the diagonal and off-diagonal terms in the effective connectivity matrix. Our model shows that reducing the self-inhibition while strengthening the lateral inhibition can substantially enhance the ability of the network to decorrelate the representations of highly similar stimuli.

### Perceptual Learning

The decorrelation of similar stimulus representations that is obtained in our model provides a natural interpretation of recent experiments on spontaneous odor discrimination via habituation [40]. Only with neurogenesis intact does enriching an animal's odor environment enhance its ability to discriminate similar odors. Since the habituation used in these discrimination experiments reflects predominantly changes in the olfactory bulb rather than higher brain areas [54]–[56], the improvement in odor discrimination resulting from odor enrichment likely reflects modifications in the encoding of the test stimuli in the olfactory bulb. Our modeling shows that fundamental features underlying the neuronal turn-over in the bulb – activity-dependent survival and reciprocal synapses – suffice to allow perceptual learning by changing the odor encoding so as to decrease their similarity and enhance their discriminability.

### Repertoire of Potentially Relevant Odors

In laboratory experiments that allow many repetitions animals can learn to discriminate highly similar odor stimuli [31], which may have highly correlated representations in the olfactory bulb. Outside the laboratory the animals are likely to face the challenge to form associations with stimuli given only a few trials. This task may be very difficult if not even impossible if the relevant odors are represented in the bulb in a highly correlated fashion.

In line with experiments on odor enrichment [40], our computational model shows that neurogenesis may facilitate this task by reducing the correlation of odors in an ensemble to which the animal is exposed. These odors could represent a repertoire of potentially relevant odor types that the animal can easily discriminate, should the need arise. In our model the survival of the granule cells depends on the inputs they receive from mitral cells via their dendro-dendritic synapses. Their relevance could be determined by the context in which the animal is exposed to the odor. Such contexts are likely to affect modulatory inputs to the olfactory bulb, which can modify the excitability of granule cells [60]–[62] and mitral cells [60], [62] as well as mitral cell inhibition [63], all of which will affect granule cell survival [64]. Contexts could also induce specific, direct inputs from cortical areas like piriform cortex to granule cells at proximal or basal synapses, both of which are functional in young granule cells with the proximal synapses developing even before the basal and dendro-dendritic ones [65], [66].

### Predictions for Effective Learning Protocols

Enrichment enhances odor discrimination only if the enrichment odors overlap in their glomerular excitation patterns with those of the test stimuli [43]. Our modeling confirms this. Moreover, it makes specific predictions with regard to the decorrelation of similar mixtures comprised of dissimilar components. If neurogenesis affects predominantly granule cells that provide lateral inhibition, our model predicts that animals will learn to discriminate such mixtures more easily if the enrichment is performed using the odor mixture rather than alternating its individual components. This difference is predicted even though both enrichment ensembles have the same overall overlap with the test stimuli. If non-topographical self-inhibition [4] should dominate neurogenetic restructuring, no difference between the protocols is expected.

The change of odor representations that our neurogenesis model predicts to arise from odor enrichment might also be testable in reward-associated discrimination tasks by focusing on the initial learning stages. Suitable enrichment protocols are expected to enhance the differences in the encoding of the test odors. Applied before the animals learn the odors that are to be discriminated, such enrichment should lead to a shortening of the initial learning phase if the odors are very similar. Moreover, it has been found that animals tend to follow different strategies during the early stages of a 2-alternative choice odor discrimination task depending on the degree of similarity of the two odors [67]. In fact, for very similar stimuli their early strategy suggests that they actually can not yet tell the test odors apart. In that case suitable prior enrichment may even allow the animals to employ their coarse-discrimination strategy for odor pairs for which without enrichment they would use the fine-discrimination strategy.

### Decorrelation by Individual and Joint Normalization

Divisive response normalization has been discussed extensively in sensory processing, in particular in the visual system [68]. In this type of normalization the response of each cell, which corresponds to a channel with given response characteristics like preferred orientation or spatial frequency of visual grid patterns, is divided by the sum of the activities of cells covering a wider range of response characteristics. The gain control implemented by this process is consistent with various experimentally observed neural responses (e.g. contrast independence and contrast adaption) [68]. In olfaction it has been proposed that such a normalization may arise from the lateral inhibition provided by the network of peri-glomerular cells, short-axon cells, and external tufted cells in the glomerular layer of the olfactory bulb [69]. Divisive normalization has been observed in the antennal lobe, which is the insect analogue of the olfactory bulb [70]. Implemented in simulations by global lateral inhibition, it was found to reduce the correlation between the different channels (activities of the principal neurons) across a large set of odors [70], [71].

Further analysis of our neurogenetic model suggests that the olfactory bulb performs a complementary type of divisive normalization (unpublished data). Rather than reshaping the mitral cell activities such that their pattern average is the essentially the same for all stimuli, the activity-dependent survival of the granule cells tends to equalize (normalize) the activity of all mitral cells when averaged across the stimulus set. Correspondingly, it foremost contributes to a reduction of the correlations between pairs of activity patterns rather than between pairs of mitral cells (channels). For stimuli whose similarity is dominated by highly co-active mitral cells the normalization of the activity of individual mitral cells achieves, however, only quite limited decorrelation. The joint normalization of the activities of multiple mitral cells, which results from the lateral inhibition of granule cells connected to multiple mitral cells, can preserve some differences in the mitral cell activities and, as a consequence, can achieve considerably better pattern decorrelation.

### Limitations of the Model

In our minimal model we have focused on the impact of the structural plasticity afforded by the turn-over of the granule cells. We have therefore treated the individual mitral and granule cells in a minimalistic fashion. In particular, we have described them in terms of linear rate dynamics without any threshold. Previous studies have shown that nonlinearities can induce stimulus decorrelation even in non-adaptive networks [3], [4], [7]. An interesting question is therefore whether neuronal nonlinearities could further enhance the decorrelation achieved by the adaptive networks studied here.

Moreover, we have modeled each neuron as a single compartment. Both, mitral and granule cells, have, however, elaborate dendrites, which most likely increase the complexity of their interaction. Thus, while action potentials can propagate with little attenuation along the mitral cell dendrite and can excite granule cells even at large distances, the inhibition that a granule cell imparts to a mitral cell is expected to depend strongly on the distance of the GABAergic synapse from the mitral cell soma. The mutual inhibition that a granule cell mediates between mitral cells will then not be symmetric. We have mimicked such an asymmetry by modifying the effective connectivity matrix and found that the network performance is quite robust with respect to such perturbations.

The dendritic computations in the granule cells are tied in with their complex multi-level calcium dynamics [50], [51]. Even quite small depolarizations of a granule cell spine can induce local GABA release, which results in graded self-inhibition of the driving mitral cell. Stronger inputs can induce low-threshold calcium spikes that can spread within the dendritic tree. Finally, suitable inputs can trigger somatically evoked conventional sodium-driven action potentials that invade the whole dendrite. This complexity may endow the granule cell with additional computational power like a dynamically regulated range of inhibition. Since the ability to generate sodium spikes develops last in adult-born granule cells [13], the balance between local signaling, calcium spikes, and sodium spikes may change with the age of the cell. While our phenomenological modeling does not capture this biophysical complexity, it shows that a reduction of the self-inhibition and a concomitant enhancement of lateral inhibition can substantially improve the decorrelation of stimulus representations.

The mechanisms controling the survival and apoptosis of granule cells are not understood in detail. It is known that larger fractions of granule cells survive if the animal is kept in odor-enriched enviroments [19] or if the excitability of the granule cells is genetically enhanced [41]. In our minimal model we therefore assumed that the survival of the granule cells increases with their activity. It has been found, however, that certain associative odor discrimination tasks can not only enhance but also reduce the survival of the adult-borne granule cells, depending on their age [23]. Recent experiments have also indicated that apoptosis of specific neurons can be elevated when associative memories are erased [72]. It would be interesting to extend our minimal model, which aims to capture the impact of neurogenesis on non-associative odor discrimination tasks [40], [44], [45], to such more complex situations.

In conclusion, using a minimal computational model we have shown that adult neurogenesis with activity-dependent apoptosis of inhibitory interneurons that are reciprocally connected with the principal neurons is sufficient to restructure a network like that of the olfactory bulb such that it learns to decorrelate representations of very similar stimuli. The network performance is quite robust with respect to various types of deviations from reciprocity that are likely to be present in the olfactory bulb. The model makes predictions regarding the impact of different enrichment protocols on the performance of animals in spontaneous and award-related odor discrimination tasks. Their outcome is expected to give insight into the type of network connectivity that is associated with the interneuronal turn-over.

## Methods

### Discrete Adaptive Network Model

We consider a minimal computational network model that focuses on the turn-over of inhibitory interneurons caused by neurogenesis and activity-dependent apoptosis and study the networks' ability to learn to decorrelate similar stimulus representations. The recurrent network comprises two types of neurons, principal neurons (mitral cells) and inhibitory interneurons (granule cells), which are coupled through reciprocal synapses (Fig. 1). Within the framework of threshold-linear rate equations the activity 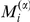 of mitral cell 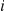 in response to stimulus 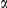 with afferent activity pattern 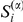, 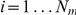, and the corresponding activity 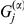 of granule cell 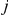, 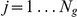, satisfy
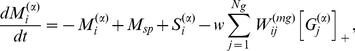
(3)

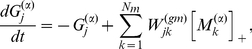
(4)with the rectifier defined as
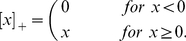
(5)Here 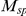 denotes the spontaneous activity of the mitral cells [46], in the absence of any odor stimulus and without any inhibitory inputs from granule cells. The stimuli 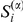 are taken from a stimulus ensemble 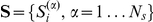. Throughout this paper we consider only the steady states that the mitral and granule cell activities reach in response to long stimulus presentations. The temporal evolution that we discuss is that of the network connectivity, which has a time scale that is much slower than that of the neuronal activities.

For strong inhibition and in particular for asymmetric connectivity matrices (cf. *RESULTS*, *Imperfect Reciprocity of Synapses is Sufficient*) the steady states of (3,4) may become unstable. While we did find complex eigenvalues in the spectrum of the linear operator of (3,4), which correspond to oscillatory modes, in none of the cases we considered did any of the eigenvalues have positive real parts. Thus, the steady output patterns remained stable for all parameters values we considered.

For sufficiently large spontaneous activity 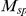 essentially all mitral cell activities – and with them the granule cell activities – are positive and the results are changed only slightly if the rectified coupling Eq.(5) is replaced by a linear coupling. We therefore use in the following only the linear coupling, which reduces the computational effort substantially.

The restructuring of the network due to neurogenesis is implemented by adding granule cells to the network at a steady rate 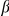 and removing them with an activity-dependent probability. No detailed information is available to what extent the formation of synapses between granule and mitral cells depends on their activity or the previous presence of synapses at that location. Since the secondary dendrites of the mitral cells, onto which the granule cells synapse, extend over large portions of the olfactory bulb we assume in this minimal model that each granule cell has the potential to establish a connection with any of the mitral cells. Thus, we assume that each of the new granule cells connects to 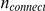 randomly chosen mitral cells. Consistent with observations [74] we assume that no granule cell connects to any mitral cell twice.

A characteristic feature of the dendro-dendritic synapses connecting mitral and granule cells is the prevalent juxtaposition of a glutamatergic synapse onto the granule cell and a GABAergic synapse onto the mitral cell [37]. There are indications that the glutamatergic and the GABAergic component form at very similar times [65], with possibly the glutamatergic component formed somehwat earlier [75]. Anatomically, the connections between these two cell types are therefore predominantly reciprocal. This reciprocity is important for the ability of the resulting network to decorrelate stimulus representations. It implies
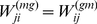
(6)with 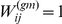 if granule cell 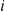 is receiving input from mitral cell 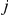 and 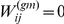 otherwise. Due to the linearity of the neuronal dynamics the effective connectivity matrix is given by
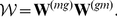
(7)Focusing on the structural plasticity provided by the persistent turn-over of granule cells rather than any plasticity of the synapses [76], [77], we assume in most of this work that all inhibitory synapses have fixed equal strength 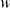 and all excitatory synapses have strength 1. To probe the role of reciprocity we consider in Sec. *RESULTS; Imperfect Reciprocity of Synapses is Sufficient* also connectivities violating (6) and heterogeneities in the synaptic strength 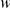.

We model activity-dependent apoptosis of the granule cells [41] by discrete events [78] during which the survival of any given granule cell is assessed based on the history of its activity. The duration of the time interval over which the activity influences cell survival is currently not known. We assume that it is long enough for the animal to be exposed to a number of relevant odors defining a stimulus ensemble **S**. Thus, at each of these events, which we assume for simplicity to occur regularly in time defining a time step of length 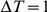, granule cells are removed probabilistically. Their survival probability 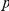 is taken to depend in a sigmoidal fashion on their cumulative, thresholded activity across the stimulus ensemble 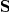. Introducing the resilience 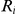 of granule cell 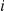 via
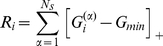
(8)with a resilience threshold 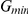, we take
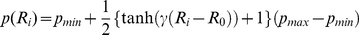
(9)with a soft survival threshold 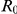. Since little is known about the specifics of the survival probability we take here 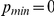 and 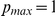. In this model a granule cell has to reach an activity beyond 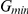 at least for some of the stimuli in the ensemble in order to trigger the signaling pathway that controls its survival [41], [57], [58].

The probabilistic network evolution eventually leads to a statistically steady state as characterized by the output patterns and their correlations fluctuating around constant values. The magnitude of the fluctuations decreases with an increase in the overall number of granule cells in the system, which can be achieved by a suitable decrease in the synaptic weight 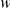. Fig. 9B shows the typical size of fluctuations in the correlation for the parameters used in our study.

The network evolution is self-regulated by the balance between proliferation and apoptosis: with increasing granule-cell population the overall inhibition of the mitral cells increases, leading to a reduction in granule cell activity. This lowers the survival probability of the granule cells and provides the saturation of the network size. As observed experimentally, reduced odor stimulation leads to a reduction in the size of the granule cell population [15].

We quantify the reshaping of the stimulus representations by the resulting network using the Pearson correlation 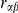 of patterns 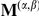


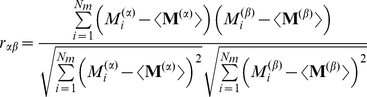
(10)where 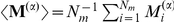. The average correlation 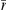 is given by
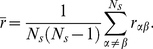
(11)


### Population Description

To capture certain aspects of the network evolution analytically we also consider the weak-coupling limit, 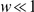. The number of granule cells is then large and the network restructuring can be described in terms of differential equations for the mean size of the various populations of granule cells that have established the same connections with mitral cells. For simplicity we give here only the equations for two-connection networks in which each granule cell makes connections with two mitral cells. The probability 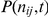 for the population of granule cells connecting mitral cells 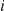 and 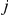 to have size 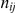 evolves during a small time step 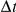 according to
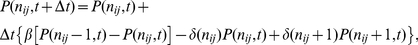
(12)where 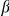 is the fixed influx of new granule cells and 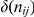 is the removal rate. With 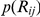 giving the probability for a granule cell to survive for the duration 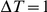, the removal rate is given by
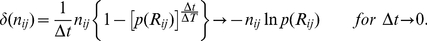
(13)Here we have used that different cells are removed independently of each other. The resilience 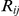 is given in terms of the activity of the granule cells 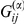 analogous to Eq.(8). For large mean population size 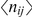 the probability distribution 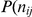,t) will be sufficiently sharply peaked to allow to approximate the evolution equation for 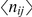,
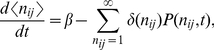
(14)by
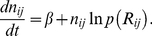
(15)Here and in the following we drop the brackets indicating the mean value.

The steady-state neuronal activities are given by
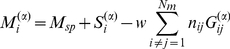
(16)

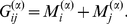
(17)


Note that for networks with a realistic number of mitral cells the number of possible different granule cell populations is extremely large, much larger than the total number of granule cells in the olfactory bulb. Thus, the size of most populations will be small and fluctuations in the number of granule cells, which have been neglected in the population description eqs.(15,16,17), may become relevant. The main purpose of the population formulation is to allow analytical approaches for simple cases, which can provide insight that may be hard to extract from numerical simulations of the discrete model. When interpreting the analytical results the limitations of the formulation need to be kept in mind.

For analytical calculations considering a steep sigmoid, 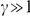, for the survival probability 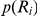 in Eq.(15) is particularly attractive. The steady state of the population description eqs.(15,16,17) can then be analyzed quite easily because the nullcline for the population 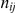, which is defined by 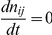, is then very well approximated by 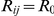 since 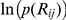 switches quickly from 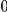 to 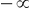 as 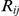 passes through 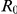.

### Interference and Optimal Resilience Threshold

To obtain analytical results for the threshold 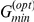 that minimizes interfering connections between mitral cells that are strongly active but only during the presentation of different stimuli we consider a set of four stimuli 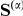 activating four glomeruli,
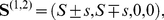
(18)

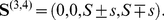
(19)The symmetry of this stimulus ensemble has been chosen such that for networks in which each granule cell connects to two mitral cells only two granule-cell populations have to be analyzed, 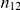 and 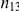. Independent of the values of the thresholds the remaining populations are given by
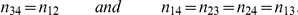
For 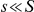 these stimulus pairs are highly correlated. We consider their reshaping by networks that are trained using the slightly simplified ensemble 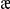 with 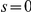. The approximate nullclines 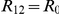 and 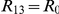 for the evolution of the two populations 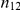 and 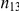 are then given by (cf. Eq.(8))
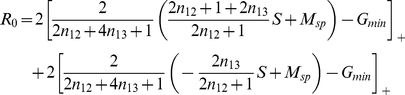
(20)

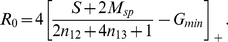
(21)Without loss of generality we have absorbed 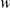 into the definition of 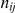. Depending on 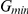 the system has two fixed points. For 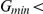 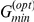 one has
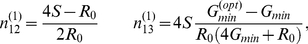
(22)where 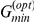 is given by
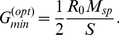
(23)For 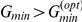 the fixed point is given by
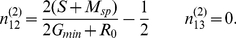
(24)Thus, the interference induced by population 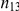 vanishes for 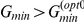, while the inhibition of the co-active cells starts to decrease at 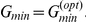 Since the correlation 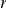 decreases with decreasing 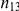 but increases with decreasing 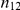 it is minimal for 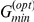.

Two comments regarding the solution (24) with 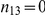 are in order. The nullclines are given by (20,21) only in the limit 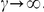 For finite values of 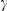 corrections arise that render 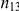 non-zero (cf. (15)). Moreover, the description of the granule cell populations solely in terms of their mean values requires that the means are sufficiently large. In particular, since the population is always non-negative its mean cannot strictly vanish. Nevertheless, for small influx 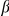 and large 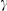 the population 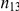 will become very small as 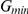 is increased beyond 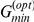.

### Alternating vs Mixture Protocol

A simple model with four stimuli and four glomeruli can also be used to obtain insight into the difference between the alternating stimulus protocol and the mixture protocol of Sec. *RESULTS; Effective Enrichment: Overall Overlap is Not Sufficient*. To mimic the alternating protocol we use stimuli (18,19) with 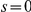 and for the training with the mixture protocol we use 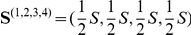. For this protocol all granule cell populations are equal, which we denote by 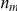.

The nullcline determining 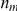 is given by
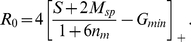
(25)For 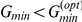 comparison with 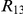 in the alternating protocol (21) gives 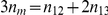, implying
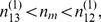
(26)where 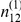 and 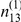 are the granule cell populations given by (22). Thus, within this simple model the mixture protocol induces stronger inhibition than the alternating protocol between the first and second pair of mitral cells, 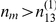. This inhibition enhances the decorrelation of stimuli like 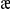 and 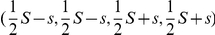, which mimic the test stimuli of Sec. *RESULT; Effective Enrichment: Overall Overlap is Not Sufficient*. This relationship among the populations is also found in the simulations of the full discrete model of Fig. 10. Excluding the terms on the diagonal, which provide self-inhibition, the sums of the synaptic weights in the four quadrants of the effective connectivity matrix 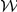 are found to be
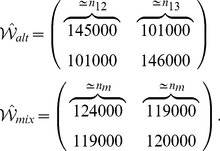
(27)The terms above the braces indicate which population is considered to correspond to which block. Note that each quadrant contains many connections that are not specific to limonene or carvone (cf. Fig. 9); neither will they be affected much by the difference in the protocol nor will they contribute substantially to the discrimination of the test stimuli.

### Natural Stimuli

To test the ability of the model network to decorrelate stimulus representations we use an ensemble of stimuli modeled after the activation patterns in the glomerular layer of rat that have been obtained experimentally via 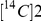-deoxyglucose uptake in response to odor exposure (published in the Glomerular Activity Response Archive http://gara.bio.uci.edu/, cf. [42]). In these data the individual glomeruli have not been identified. Clearly, not each of the 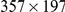 pixels corresponds to a glomerulus. We have down-sampled the experimentally determined pixel patterns to 424 input channels (or 50 channels in cases in which we illustrate the connectivity), and take each channel as a proxy for a glomerulus. In the down-sampling we avoid excessive smoothing of the resulting patterns by retaining in each set of adjacent 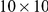 pixels the highest value rather than their average (Fig. 2A). The stimulus set 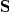 includes 2 pairs of enantiomers, 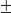-limonene and 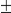-carvone, which are difficult to discriminate. Specifically, without training mice do not discriminate between the two enantiomers of limonene [40]. When addressing the ability of the model network to learn to decorrelate highly similar stimuli we focus on these 4 stimuli. In addition, to mimic a background odor environment we include four additional stimuli, 1-butanol, 1-hexanol, 1-heptanol, and acetic acid.

## Supporting Information

Text S1 (with Figs.S1,S2,S3) Comparison of the decorrelation performance of the neurogenetic network with that of other adaptive networks.
(PDF)
