# Supplementary material for: Neurogenesis Drives Stimulus Decorrelation in a Model of the Olfactory Bulb
Source: PLoS Comput Biol. 2012 Mar 15;8(3):e1002398. doi: 10.1371/journal.pcbi.1002398 (PMC3305347; doi:10.1371/journal.pcbi.1002398)
Supplement: Text S1 — (with Figs.S1,S2,S3) Comparison of the decorrelation performance of the neurogenetic network with that of other adaptive networks. (PDF) [file pcbi.1002398.s001.pdf]

Supporting Information: Neurogenesis Drives Stimulus Decorrelation in a Model of the Olfactory Bulb

Siu-Fai Chow[1], Stuart D. Wick[3], Hermann Riecke[1,2,*]

1 Engineering Sciences and Applied Mathematics, Northwestern University, Evanston, IL 60208, USA
2 Northwestern Institute on Complex Systems, Northwestern University, Evanston, IL 60208, USA
3 Department of Physics, North Central College, Naperville, IL 60540, USA
∗ E-mail: Corresponding h-riecke@northwestern.edu

Comparison of Different Types of Adaptive Networks

Various types of adaptive networks have been discussed in the context of stimulus discrimination by the olfactory bulb. In an early model for the restructuring of the inhibitory bulbar network by neurogenesis granule cells were not treated explicitly. Instead, inhibition was assumed to be pairwise and symmetric between mitral cells with a strength that was taken as a proxy for the number of granule cells connecting the two mitral cells [1]. The mitral cells were described with a linear firing-rate model. The inhibitory weight of the connection between two mitral cells was increased proportional to the pairwise scalar product of the activity of these two mitral cells across a stimulus ensemble. Since the activities represented deviations from the mean they could also be negative in this model.

From a more general perspective it has been shown that a network can make stimulus representations orthogonal if it normalizes the activity of each output channel (i.e. the activity of each mitral cell) across the stimulus ensemble and orthogonalizes the output activities [2]. In a recurrent inhibitory network with symmetric connectivity this is achieved if the inhibition between the channels is essentially given by the square-root of the matrix of pairwise scalar products of the input activities.

For the honey bee olfactory system experimental data are available for the inputs as well as the outputs of the antennal lobe. These were employed in a computational model of the antennal lobe to show that the pattern transformation performed by the antennal lobe is better captured by models in which the connectivity of the inhibitory network is based on the correlations between the glomerular inputs than by models with random or spatially local connectivity [3].

Motivated by these different types of adaptive networks we compare the ability of a number of different adaptive recurrent networks to decorrelate the natural stimuli of Fig.2. In all cases only the principal neurons are retained and their dynamics are given by the linear rate model

$$\frac{dM_i^{(\alpha)}}{dt} = -M_i^{(\alpha)} + M_{sp} + S_i^{(\alpha)} - w \sum_{j=1}^{N_m} \mathcal{W}_{ij} M_j^{(\alpha)} \tag{1}$$

with an effective inhibitory connectivity matrix $\boldsymbol{\mathcal{W}}$. We use $\mathbf{S} = \{S_i^{(\alpha)},\ \alpha = 1 \dots N_s\}$ to denote the ensemble of N_s stimuli in terms of a matrix in which the columns consist of the stimulus activities. In our neurogenetic model the spontaneous mitral cell activity M_{sp} modifies the connectivity. In the other connectivities we incorporate M_{sp} by using an effective stimulus ensemble $\hat{\mathbf{S}} = \mathbf{S} + \mathbf{M}_{sp}$, where $\mathbf{M}_{sp}$ is a

matrix in which all entries are equal to M_{sp}. The connectivity matrices are then given by

$$\mathcal{W}_{ij}^{(ng)} = \mathbf{W}^{(mg)}\mathbf{W}^{(gm)} \quad \text{Neurogenesis} \tag{2}$$

$$\boldsymbol{\mathcal{W}}^{(ortho)} = \left[\frac{1}{\Gamma}\left(\hat{\mathbf{S}}\hat{\mathbf{S}}^t\right)^{\frac{1}{2}} - \mathbf{I}\right]_+ \quad \text{Orthogonalizing} \tag{3}$$

$$\boldsymbol{\mathcal{W}}^{(L_2)} = \hat{\mathbf{S}}\hat{\mathbf{S}}^t \quad \text{L}_2 \tag{4}$$

$$W_{ij}^{(dist)} = \frac{1}{\sqrt{\sum_{\alpha=1}^{N_s}\left(S_i^{(\alpha)} - S_j^{(\alpha)}\right)^2}} \quad \text{Distance} \tag{5}$$

$$W_{ij}^{(corr)} = \left[r(S_i, S_j)\right]_+ \quad \text{Pearson correlation} \tag{6}$$

$$W_{ij}^{(cov)} = \left[\text{cov}(S_i, S_j)\right]_+ \quad \text{Covariance.} \tag{7}$$

In each of these connectivities the inhibition between pairs of mitral cells is related to a broadly defined similarity of their responses to the stimuli in the ensemble. The similarity is, however, assessed in different ways in each case. Connectivity $\boldsymbol{\mathcal{W}}^{(ng)}$ is obtained from our neurogenesis model (eqs. (3,4,9) in the main manuscript) with 50 mitral cells. In this model the similarity is measured in terms of an additive co-activity. In connectivities $\boldsymbol{\mathcal{W}}^{(ortho)}$ and $\boldsymbol{\mathcal{W}}^{(L_2)}$ the similarity is measured in terms of a multiplicative co-activity. The connectivity $\boldsymbol{\mathcal{W}}^{(ortho)}$ stems from the orthogonalizing networks [2], with the parameter Γ determining the L_2-norm of the outputs with $w = 1$ fixed. Without any attempt to optimize the performance we choose here $\Gamma = 1$. Connectivity $\boldsymbol{\mathcal{W}}^{(L_2)}$ is motivated by the algorithm used in [1]. Connectivity $\boldsymbol{\mathcal{W}}^{(corr)}$ is motivated by the modeling of the antennal lobe network [3]; here $r(S_i, S_j)$ denotes the Pearson correlation between the stimulus activities of glomerulus i and glomerulus j given the stimulus ensemble $\mathbf{S}$. Analogously, in connectivity $\boldsymbol{\mathcal{W}}^{(cov)}$ $\text{cov}(S_i, S_j)$ denotes the corresponding covariance. Writing $\mathbf{S}$ instead of $\hat{\mathbf{S}}$ in $\boldsymbol{\mathcal{W}}^{(corr)}$, $\boldsymbol{\mathcal{W}}^{(cov)}$, and $\boldsymbol{\mathcal{W}}^{(dist)}$ emphasizes the fact that these connectivities are not sensitive to M_{sp}. The connectivity matrices $\boldsymbol{\mathcal{W}}^{(ortho)}$, $\boldsymbol{\mathcal{W}}^{(corr)}$, and $\boldsymbol{\mathcal{W}}^{(cov)}$ are not guaranteed to have only positive entries. We therefore set any negative entries to 0.

To compare the different connectivities we use two different approaches. Since the decorrelation performance often improves with increasing inhibition and decreasing output amplitude, we choose the overall inhibitory weight w for each connectivity such that it generates the same mean output amplitude as the neurogenesis model. Some connectivities perform, however, optimally at an intermediate inhibitory strength. Therefore we also consider the dependence of the performance on the inhibitory strength.

We characterize the different networks in two ways. We determine the similarity of the connectivities directly using a scaled distance $\mathcal{D}$ between them, which we define as

$$\mathcal{D}(\mathbf{W}^{(1)}, \mathbf{W}^{(2)})^2 = \frac{\sum_{ij}\left(W_{ij}^{(1)} - W_{ij}^{(2)}\right)^2}{\frac{1}{2}\left(\sqrt{\sum_{ij} W_{ij}^{(1)2}} + \sqrt{\sum_{ij} W_{ij}^{(2)2}}\right)^2}. \tag{8}$$

Then we assess the performance of the networks in terms of the correlations of their outputs given the stimulus ensemble $\mathbf{S}$.

The distance measure $\mathcal{D}$ reveals that the co-activity based connectivities $\boldsymbol{\mathcal{W}}^{(ng)}$, $\boldsymbol{\mathcal{W}}^{(ortho)}$, and $\boldsymbol{\mathcal{W}}^{(L_2)}$ are quite similar to each other for both values of M_{sp} (Fig.S1). The relationship among the other connectivities is not as clear. For $M_{sp} = 0$ it appears as if $\boldsymbol{\mathcal{W}}^{(dist)}$ and $\boldsymbol{\mathcal{W}}^{(corr)}$ also formed a cluster. However, it does not persist for $M_{sp} = 1$ (Fig.S1B) and other values of the mean output amplitude (not shown). Similarly, for some output amplitudes $\boldsymbol{\mathcal{W}}^{(corr)}$ and $\boldsymbol{\mathcal{W}}^{(cov)}$ are much closer to each other than for the output amplitudes used in Fig.S1.

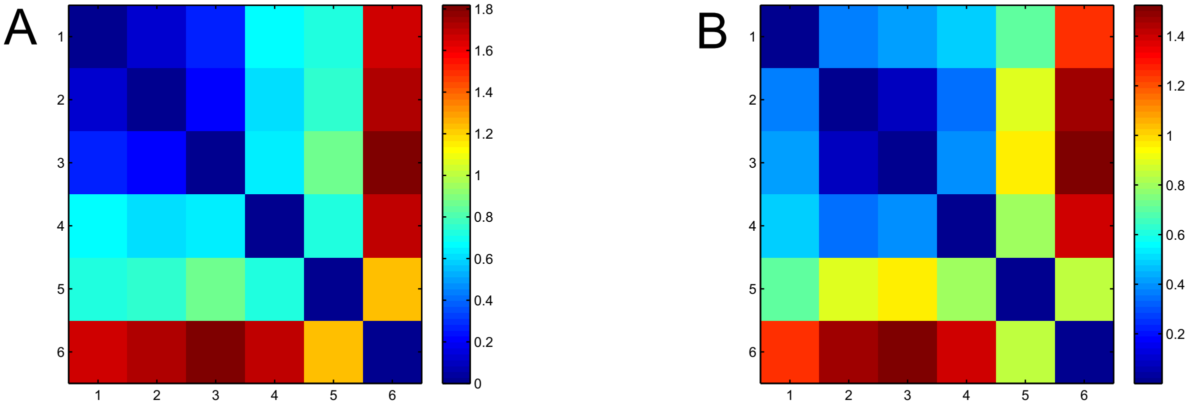

Figure S 1. Similarity of connectivities. Matrix of scaled pairwise distance $\mathcal{D}$ between the connectivities (2-7) for $M_{sp} = 0$ with coupling strengths $\mathbf{w} = (0.002, 1.35, 0.32, 0.13, 0.85, 70)$ (**A**) and $M_{sp} = 1$ with coupling strengths $\mathbf{w} = (0.002, 0.31, 0.012, 0.105, 0.8, 50)$ (**B**). In the distance matrix and the list of coupling strengths $\mathbf{w}$ the order of the connectivities is $\mathcal{W}^{(ng)}$, $\mathcal{W}^{(ortho)}$, $\mathcal{W}^{(L_2)}$, $\mathcal{W}^{(dist)}$, $\mathcal{W}^{(corr)}$, $\mathcal{W}^{(cov)}$. With these synaptic weights w the mean output amplitude is the same for all connectivities, $\bar{\bar{M}} \equiv (N_s N_m)^{-1} \sum_{i,\alpha} M_i^{(\alpha)} = 0.0265$ for $M_{sp} = 0$ and $\bar{\bar{M}} = 0.15$ for $M_{sp} = 1$. For $\mathcal{W}^{(cov)}$ and $M_{sp} = 1$ the output amplitude had to be chosen larger, $\bar{\bar{M}} = 0.189$, to avoid dynamical instability in (1). Parameters for the runs generating $\mathcal{W}^{(ng)}$: $N_m = 50$, $\gamma = 20$, $R_0 = 0.1$, $n_{connect} = 4$, $\beta = 3.9$, $w = 0.002$, **A:** $M_{sp} = 0$, $G_{min} = 0.12$, **B:** $M_{sp} = 1$, $G_{min} = 0.6$.

To assess the decorrelation performance of the networks directly we determine the correlations in the outputs for the stimuli to which they are adapted. Without the spontaneous activity of the mitral cells, $M_{sp} = 0$, each of the connectivities (2-7) is able to reduce the correlation of the representations of the highly similar stimuli (black bars in Fig.S2A), albeit to very different degrees. This is not the case for the less correlated stimuli. Consistent with the similarity of the co-activity based connectivities seen in Fig.S1, $\mathcal{W}^{(ng)}$, $\mathcal{W}^{(ortho)}$, and $\mathcal{W}^{(L_2)}$ perform similarly and quite well for all stimuli. The connectivities $\mathcal{W}^{(dist)}$, $\mathcal{W}^{(corr)}$, and $\mathcal{W}^{(cov)}$, however, only decorrelate the representations of the highly similar stimuli; for the less related inputs their performance is very poor; in fact, on average the outputs of $\mathcal{W}^{(corr)}$ and $\mathcal{W}^{(cov)}$ are more correlated than their inputs (Fig.S2B).

For non-zero spontaneous activity, $M_{sp} = 1$, most connectivities perform worse, in particular for the highly similar stimuli. Nevertheless, the co-activity based connectivities perform still quite well overall. The performance of $\mathcal{W}^{(corr)}$, $\mathcal{W}^{(cov)}$, and $\mathcal{W}^{(dist)}$, however, is poor. Even the representations of the highly similar stimuli are not decorrelated any more. This reflects, in part, the fact that these adaptation schemes do not account for the spontaneous mitral cell activity, which effectively corresponds to a significant mean value in the input activities.

The strikingly poor decorrelation by the correlation-based and the covariance-based network for the inhibition level used in Fig.S2 raises the question whether their performance could be improved by optimizing the overall inhibition. We therefore vary for each network the overall inhibition level w while keeping the network structures fixed as defined by (2-7). For adaptive networks the inhibition level w affects, in principle, also the training of the network; the network structure is therefore not really independent of w. However, none of the comparison networks (3-7) are defined via a training algorithm; for them w is a free parameter. Varying w over a range that induces comparable changes in the output

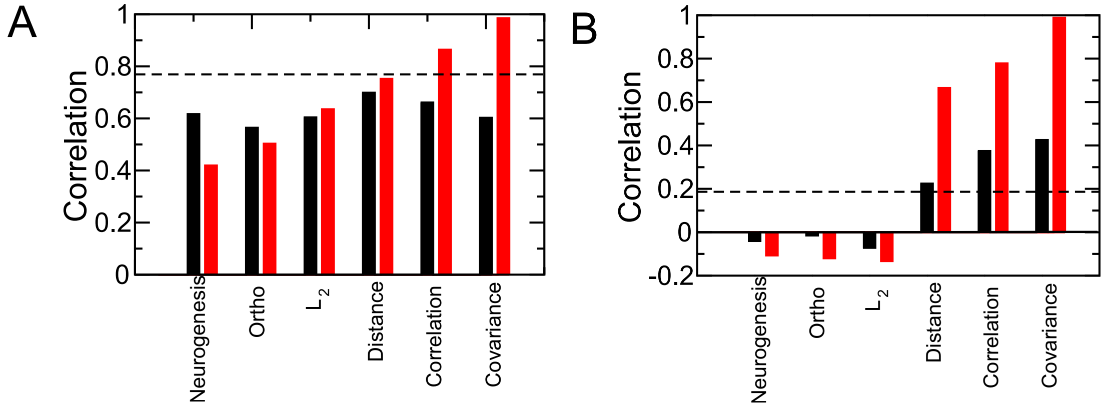

Figure S 2. Comparison of the decorrelation performance of adaptive networks. A) Top correlations $r^{(top)}$ obtained with the connectivities (2-7). **B)** Mean correlations $\bar{r}$ obtained with the connectivities (2-7). $M_{sp} = 0$ (black), $M_{sp} = 1$(red). Dashed lines denote the corresponding input correlations.

amplitude we find that the performance of the co-activity based connectivities improves monotonically with decreasing amplitude. In Fig.S3 this is shown for the top correlation $r^{(top)}$ for $M_{sp} = 0$ (black) and $M_{sp} = 1$ (red). It also holds for the overall correlation (not shown). The connectivities $\mathcal{W}^{(corr)}$ and $\mathcal{W}^{(cov)}$ exhibit different behavior. For $M_{sp} = 0$ they both decorrelate the representations of the highly similar stimuli for weak inhibition, but as the inhibition is increased the output correlation starts to rise again. With non-zero spontaneous mitral cell activity the output correlation increases monotonically already starting from $w = 0$. We find that the overall correlation $\bar{r}$ increases monotonically even for $M_{sp} = 0$ (not shown). The distance-based connectivity reduces $r^{(top)}$ monotonically for $M_{sp} = 0$. For $M_{sp} = 1$, however, the correlation increases to a maximum before it starts to decrease. Similarly, the overall correlation $\bar{r}$ increases initially with increasing inhibition.

The network $\mathcal{W}^{(ortho)}$ is designed to orthogonalize the representation of the stimulus ensemble. Nevertheless, in the situations shown in Fig.S2A and Fig.S3A it does not achieve this goal for the highly similar stimuli. This is a result of the requirement that the network be purely inhibitory, i.e. $W_{ij}^{(ortho)} \geq 0$. Depending on the stimulus ensemble and the desired output amplitudes this restriction limits its performance to a variable degree. In other situations essentially perfect decorrelation is obtained [2].

Our investigation of connectivities $\mathcal{W}^{(L_2)}$ and $\mathcal{W}^{(corr)}$ is motivated by previous work on neurogenesis [1] and odor processing in the antennal lobe [3]. It should be noted, however, that these connectivities do not in detail represent the networks investigated in [1, 3]. In [1] the effective weights are updated according to the L_2 scalar product of the mitral cell activities and additional self-inhibition as well as a total synaptic weight normalization is introduced. In [3] the inhibition is feedforward rather than recurrent, i.e. it is driven directly by the inputs.

This survey does not aim to represent an exhaustive analysis of the various decorrelation mechanisms. It suggests, however, that co-activity based connectivities like $\mathcal{W}^{(ng)}$, $\mathcal{W}^{(ortho)}$, and $\mathcal{W}^{(L_2)}$ are much more capable to decorrelate stimulus representations under various conditions than correlation- or covariance-based networks.

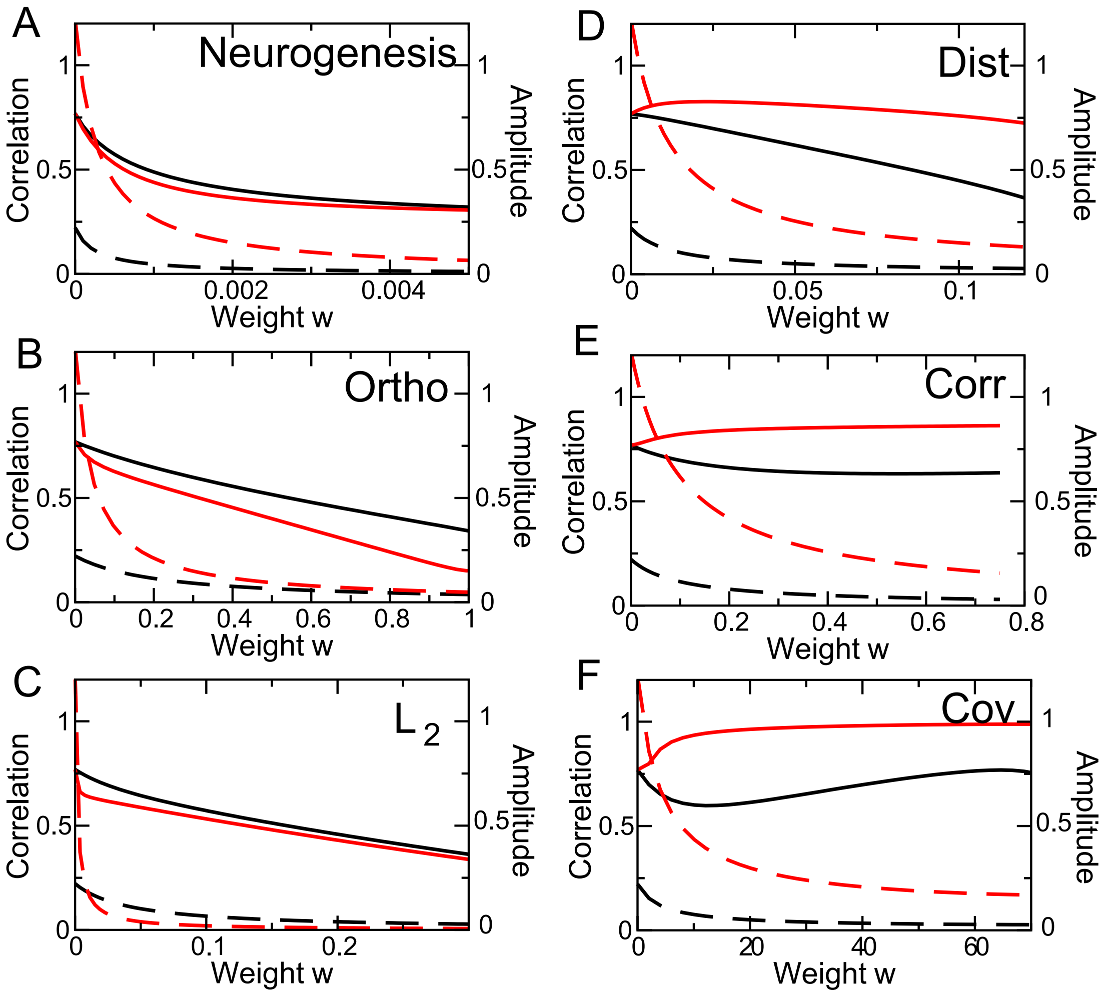

Figure S 3. Dependence of the decorrelation of the highly similar stimuli on the overall weight w. Black: $M_{sp} = 0$, red: $M_{sp} = 1$. Solid lines $r^{(top)}$, dashed lines: mean amplitude.

References

1. Cecchi GA, Petreanu LT, Alvarez-Buylla A, Magnasco MO (2001) Unsupervised learning and adaptation in a model of adult neurogenesis. J Comput Neurosci 11: 175-182.

2. Wick S, Wiechert M, Friedrich R, Riecke H (2010) Pattern orthogonalization via channel decorrelation by adaptive networks. J Comput Neurosci 28: 29–45.

3. Linster C, Sachse S, Galizia CG (2005) Computational modeling suggests that response properties rather than spatial position determine connectivity between olfactory glomeruli. J Neurophysiol 93: 3410-3417.
